# Rubella in Sub-Saharan Africa and sensorineural hearing loss: a case control study

**DOI:** 10.1186/s12889-017-4077-2

**Published:** 2017-02-01

**Authors:** Cristina Caroça, Vera Vicente, Paula Campelo, Maria Chasqueira, Helena Caria, Susana Silva, Paulo Paixão, João Paço

**Affiliations:** 1 Otolaryngology Department, NOVA Medical School/Faculty of Medical Sciences, Universidade Nova de Lisboa, Campo dos Mártires da Pátria, 130, 1169-056 Lisboa, Portugal; 2 Hospital CUF Infante Santo, Avenida Infante Santo, 34, 6°, 1350-079 Lisboa, Portugal; 3 Centre for Toxicogenomics and Human Health (ToxOmics), NOVA Medical School / Faculty of Medical Sciences, Universidade Nova de Lisboa, Campo dos Mártires da Pátria, 130, 1169-056 Lisboa, Portugal; 4 CEDOC, NOVA Medical School/ Faculty of Medical Sciences, Universidade Nova de Lisboa, Lisboa, Portugal; 5 Escola Superior de Tecnologia da Saúde de Lisboa, Avenida D. João II, Lote 4.69.01, 1990-096 Lisboa, Portugal; 6 BioISI-Biosystems and Integrative Sciences Institute, Faculty of Science of the University of Lisbon, Lisbon, Portugal; 7 ESS/IPS, School of Health, Polytechnic Institute of Setúbal, Setúbal, Portugal; 8 Clinical Pathology Laboratory—Labco, Hospital da Luz, Avenida Lusíada, 100, 1500-650 Lisboa, Portugal

**Keywords:** Rubella, Hearing loss, Sub-Saharan Africa, Congenital Rubella Syndrome, World Health Organization

## Abstract

**Background:**

Rubella infection can affect several organs and cause birth defects that are responsible for congenital rubella syndrome (CRS). Congenital hearing loss is the most common symptom of this syndrome, occurring in approximately 60% of CRS cases. Worldwide, over 100 000 babies are born with CRS every year. There is no specific treatment for rubella, but the disease is preventable by vaccination. Since 1969, the rubella vaccine has been implemented in many countries, but in Africa, only a few countries routinely immunize against rubella. The aim of this study was to estimate the rate of infection from the wild-type rubella virus in São Tomé and Príncipe by determining rubella seroprevalence with a DBS method. The goal of this study was to reinforce the need for implementation of the rubella vaccine in this country. As secondary objectives, the validation of a DBS method was first attempted and an association between seroprevalence and hearing loss was assessed.

**Methods:**

We collected samples from individuals observed during humanitarian missions in São Tomé and Príncipe. All individuals underwent an audiometric evaluation, and a drop of blood was collected for the dried blood spot (DBS).

We define two groups: the case group (individuals with unilateral or bilateral hearing loss (HL)) and the control group (individuals with two normal ears). Patients were excluded if they suffered from conductive HL, if they showed evidence of possible causes of HL, if they had developmental delay or if they refused to participate in the study.

**Results:**

Among the 315 subjects, we found 64.1% individuals with IgG for the rubella virus, 32.1% without immunity for the rubella virus and 3.8% who were borderline.

In the control group, 62.6% were positive for the rubella IgG, whereas in the case group, 72% were positive. Analyzing both groups, with ages ranging from 2 to 14 years of age and from 15 to 35 years of age, we found a seroprevalence of 50.3% to rubella in the younger group and 82.1% in the older group, with a significant difference between cases and control group noted within the younger patients (*p* = 0.025).

**Conclusions:**

Rubella is a disease that can be prevented. Rubella infections are still very common in São Tomé and Príncipe, and women of child-bearing age are still at risk for rubella infection during pregnancy, justifying the urgency of vaccination against rubella.

A statistically significant association between the group of children under 14 years of age with HL and immunity for rubella was observed in this country, although this study did not allow us to establish a cause-effect relationship between rubella infection and SNHL.

## Background

Primary rubella infection during pregnancy, particularly during the first trimester, can affect several organs and cause birth defects that are responsible for congenital rubella syndrome (CRS) [1]. The most common defects of CRS are hearing impairment (unilateral or bilateral sensorineural), eye defects (e.g., cataracts, congenital glaucoma, or pigmentary retinopathy), and cardiac defects (e.g., patent ductus arteriosus or peripheral pulmonic stenosis). Congenital hearing loss is the most common sequela, occurring in approximately 60% of cases, especially when infection occurs in the 4th month of pregnancy [2]. In a Brazilian study, congenital rubella was thought to be the cause of hearing loss in 32% of patients with deafness [3], and in studies conducted in sub-Saharan Africa, rubella was considered to be one of the causes of HL [4].

The Global Measles and Rubella Strategic Plan (2012–2020) included goals to eliminate rubella and CRS in at least two WHO regions by 2015 as well as in at least five WHO regions by 2020. However, in this plan, the African region does not have a specific target. The number of rubella cases reported from 2000 to 2014 increased in the African region (from 865 cases in seven countries to 7402 cases in 44 countries). Although the rubella vaccine has been implemented in many countries since 1969, worldwide coverage is still a distant goal, particularly in Africa, where only a few countries routinely immunize against rubella [5, 6].

The efficacy of the vaccine is approximately 95%, without significant side effects [7]. In 2015, the Americas region was the world’s first region to eliminate rubella and CRS. In Europe, all 53 Member States of the WHO European Region committed in 2010 to the goal of interrupting the endemic transmission of measles and rubella by 2015, which was not yet achieved in all regions. However, many states, including Portugal, have already interrupted the endemic transmission of rubella [8].

The São Tomé and Príncipe islands are within the Atlantic in sub-Saharan Africa, located at the level of the Equator. It is an underdeveloped country with few economic resources that survives through external support, including humanitarian service. As part of a humanitarian project on the islands of São Tomé and Príncipe, the “Health for All” system of Institute Marquês Valle Flor (IMVF) has been implemented to improve the primary and secondary health care of the population. This project includes doctors of several specialties, including otolaryngology.

During these tasks, an increased prevalence of sensorineural hearing loss (SNHL) cases was initially noted, particularly in the younger age group [9]. CRS cannot be excluded as a possible etiology of SNHL and is one of several possible causes.

The epidemiology of rubella is not known in this country, and there is no vaccine implementation [10] nor is there the possibility of diagnosis through laboratory tests.

The aim of this study is to estimate the rate of infected people with wild-type rubella virus in São Tomé and Príncipe by determining rubella seroprevalence through the DBS method to reinforce the need for vaccine implementation in this country. As secondary objectives, the validation of a DBS method was first attempted and an association between seroprevalence and hearing loss was also evaluated.

## Methods

### Subjects

The samples studied were collected between January and May of 2014 from individuals who presented for an audiology consultations at the Hospital Ayres de Menezes on São Tomé Island and the Hospital Dr Manuel Quaresma Dias da Graça on Principe Island during humanitarian missions. Samples were also collected from students and workers from a hotel. All participants in the study were natives and residents of the islands. In total, we analyzed 315 samples collected from individuals 2 to 35 years old. Of these, 171 individuals were female and 144 were male. All individuals underwent audiometric evaluation (tonal audiogram or auditory brainstem response measurements) according to the degree of collaboration, having adapted the results of the auditory brainstem response (ABR) to audiometric thresholds according to standards [11].

We defined two groups based on the WHO classification [12]. The case group was composed of individuals with hearing loss, in which we included individuals with unilateral HL or both ears with HL. The control group included individuals with two normal ears.

All patients answered a questionnaire about self-reported HL and clinical history. There are no clinical registries about these patients in any hospital from São Tomé or Príncipe.

Patients were excluded if they suffered from conductive deafness, showed evidence of possible causes of HL, had developmental delay, or did not give consent to participate in the study.

In addition to the audiometric evaluation, a drop of blood was collected via venous or capillary puncture and blotted onto filter paper. After collection, the blood spots were dried at room temperature for 24 h (Dried blood spot—DBS). The IgG measurement was carried out in Portugal after 9 months (during which the samples were stored at room temperature).

The project was submitted to and approved by the Medical Ethics Committee of STP and Ethics Research Committee NMS|FCM-UNL (n°02/2014/CEFCM). The Ethics Research Committee is aligned with the Declaration of Helsinki for the Protection of Human Subjects. A full consent process was applied for all participants. Consent to use the survey data was also obtained.

### Technical validation of the rubella IgG determination

The procedure of IgG determination from DBS was validated and optimized by two approaches: 1) First, samples were collected from 20 pregnant women at the Hospital da Luz, Portugal. We simultaneously collected blood samples for DBS and for serum. The specificity and sensitivity of the IgG determination from DBS were evaluated compared to the standard method (IgG determination from serum). 2) Second, 15 DBS samples were collected in 10 children between 1 and 10 years of age (who were vaccinated against rubella) and 5 children between 9 and 12 months of age (who were not vaccinated against rubella). In this group, IgG determination from DBS was correlated with the immune status (vaccinated / unvaccinated).

The extraction protocol for IgG in DBS was tested with three different volumes of diluent (200 μL, 400 μL, and 800 μL) in this validation step, while the protocol for the determination of the serum IgG was recommended by the SERION ELISA classic rubella virus IgG kit. Both the extraction and IgG ELISA protocols are described below.

### Rubella IgG determination from the São Tomé and Príncipe population

Rubella IgG extraction: For the IgG extraction, we added 400 μL of SERION ELISA kit dilution solution to ¼ of the DBS, corresponding to 32 mm^2^. The extraction was carried out for 60 min at 600 rpm at room temperature and 18 h at 4 °C.

Rubella IgG determination: The SERION ELISA classic rubella virus IgG kit was used for this determination. We performed the protocol recommended by SERION. Briefly, 100 μL of the control, standard and extracted samples were pipetted into a 96 well microplate (only one sample per patient was tested). The microplate was then incubated at 37 °C for 60 min in a humid chamber and washed 3 times with wash solution (300 μl). Then, the IgG conjugate (100 μl) was added and the microplate was incubated under the same conditions, after which the washing process was repeated. Subsequently, the substrate (100 μl) was added and the incubation process was repeated. Finally, we added a stop solution (100 μl), and the optical density was read at 415 nm against 630 nm. The optical densities were converted into UI through the Serion Activity V11 program, with the following interpretation: negative: <10UI; borderline; 10–15UI;positive >15UI.

**Fig. 1:**
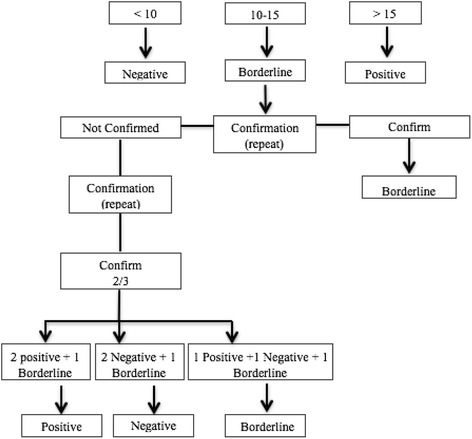
Algorithm for the interpretation of the results. The results are expressed in international units (U/I)

The results were interpreted according to the algorithm shown in Fig. 1.

### Statistical processing of the data

Sample descriptions were made using descriptive statistics, considering the frequency analysis, means and standard deviations (SDs).

To study the association between IgG rubella in the case/ control group and each of the following parameters, age group, district origin, oral language, gender and HL, the chi-square test was used by Monte Carlo Simulation.

To identify the risk factors of HL, we adopted a Binary Logistic Regression, where HL is a response variable. The independent variables were the IgG rubella and age groups.

All analyses were performed using the Statistical Package for the Social Sciences for Mac version 20.0 (SPSS).

## Results

### Technical validation of the rubella IgG determination

The sensitivity and specificity for each of the different volumes of diluent tested, when compared with the standard method were, 100 and 50% (200 μL), 89 and 100% (400 μL) and 72 and 100% (800 μL). According to these results, a volume diluent of 400 μL was chosen for the determination of Rubella IgG in the São Tomé and Príncipe populations.

The DBS method also showed a good correlation (100%) with the immune status (vaccinated/unvaccinated). The ten vaccinated children were positive for the IgG while the five unvaccinated children were negative for IgG.

### Rubella IgG determination from São Tomé and Príncipe population

We evaluated 315 subjects (Table 1), from 2 to 35 years of age, of whom 144 (45.7%) were men and 171 (54.3%) were women, with a mean age of 17.36 ± 9.734 years.

**Table 1:** General characteristics of the São Tomé and Príncipe population

	Total (*n* = 303)	Control Gr (171–56.4%)	Case Gr (132–43.6%)	*p-Value*
Age range				*0.359*
[2–14] [15–35]	**147 (48.5%)** **156 (51.5%)**	79 (46.2%) 92 (53.8%)	68 (51.5%) 64 (48.5%)	
Mean Age SD	**17.31 ± 9.722**	17.73 ± 9.729	16.77 ± 9.723	
Gender				*0.391*
Male Female	**137 (45.2%)** **166 (54.8%)**	81 (47.4%) 90 (52.6%)	56 (42.4%) 76 (57.6%)	
Resident District				0.061*
Água Grande Mezochi Cantagalo Caué Lemba Lobata Príncipe	**174 (57.4%)** **47 (15.5%)** **17 (5.6%)** **23 (7.6%)** **6 (2%)** **19 (6.3%)** **17 (5.6%)**	110 (64.3%) 17 (9.9%) 8 (4.7%) 12 (7%) 3 (1.8%) 11 (6.4%) 10 (5.8%)	64 (48.5%) 30 (22.7%) 9 (6.8%) 11 (8.3%) 3 (2.3%) 8 (6.1%) 7 (5.3%)	
Spoken Language				***0.0001***
Yes No Undefined	**242 (86.7%)** **37 (13.3%)** **24**	161 (97.6%) 4 (2.4%) 6	81 (71.1%) 33 (28.9%) 18	
Family History of HL				*0.207*
Yes No Missing	**52 (17.5%)** **245 (82.5%)** **6**	33 (20%) 132 (80%) 6	19 (14.4%) 113 (85.6%)	
Consanguinity				*0.461*
Yes No Missing	**7 (2.4%)** **285 (97.6%)** **11**	3 (1.8%) 162 (98.2%) 6	3 (3.1%) 123 (96.9%) 5	
Rubella IgG				*0.085*
Positive Negative Borderline	**202 (66.7%)** **101 (33.3%)** **12**	107 (62.6%) 64 (37.4%)	95 (72%) 37 (28%)	

*Abbreviations*: *SD* standard deviation

*Chi-square Test by Monte Carlo Simulation; Bold: Total sample; Italic: *p*-Value; Italic bold: statistic significant *p*-Value

Among the 315 subjects, we found 202 (64.1%) individuals with IgG for the rubella virus. Of these, 101 (32.1%) did not have immunity to rubella and 12 (3.8%) were borderline. Borderline cases were excluded from the study.

In the sample, we did not find any statistical significance for gender (*p* = 0.391), resident district (*p* = 0.061, or chi-squared test by Monte Carlo simulation), family history of HL (*p* = 0.207), or consanguinity (*p* = 0.461).

We established two groups concerning hearing status: a control group with patients with normal hearing in both ears and a case group with at least one ear with sensorineural hearing loss (SNHL).

In the control group, we found 62.6% of positive immunity to rubella, whereas in the case group, positive immunity was 72%. There was not a significant difference between the case and control groups (*p* = 0.085).

Analyzing both the 2-to-14 years of age group and the 15-to-35 years of age group, we found a seroprevalence of 50.3% to rubella in the younger group and 82.1% in the older group, with a significant difference between the case and control groups within the younger group (*p* = 0.025) but not within the older group (*p = 0.528*) (Table 2).

**Table 2:** Sample description of the case/control group and rubella IgG in the different age ranges

	Total (*n* = 303)	IgG Pos (117–58.8%)	IgG Neg (82–41.2%)	*p-Value*
Age range				***0.025***
[2–14]	**147 (48.5%)**	74	73	
Control group Case group	79 68	33 (44.6%) 41 (55.4%)	46 (63%) 27 (37%)	
Age range				*0.528*
[15–35]	**156 (51.5%)**	128	28	
Control group Case group	92 64	74 (57.8%) 54 (42.2%)	18 (63.3%) 10 (35.7%)	

Bold: Total sample; Italic: *p*-Value; Italic bold: statistic significant *p*-Value

By applying the binary logistic regression model, positive immunity to rubella was identified as a risk factor for HL (Table 3). Positive immunity to rubella almost doubled the risk of HL (OR = 1.776; CI 95% [1.050–3.004]) when analyzed with the age groups, but without any statistical significance in these age groups. Therefore, immunity to rubella was associated with HL.

**Table 3:** Binary Logistic Regression between Rubella and HL with and without factors (age group)

	Cases *n* (%)	Controls *n* (%)	*P*-value	Crude OR (95% CI)	*P*-value	Adjusted OR (95% CI)
Age group						
			*0.359*		*0.111*	
[2–14] [15–35]	68 (51.5%) 64 (48.5%)	79 (46.2%) 92 (53.8%)		Reference 0.808 [0.513–1.274]		Reference 0.672 [0.412–1.096]
Rubella IgG						
			*0.086*		***0.032***	
Negative	37 (28%)	64 (37.4%)		Reference		Reference
Positive	95 (72%)	107 (62.6%)		1.536 [0.941–2.507]		1.776 [1.050–3.004]

*Abbreviations*: *HL* normal hearing – 0 – Reference Category is a response variable, *independent variables* Rubella IgG (0 –Negative, 1- Positive), age groups (0 – [2–14] years, 1 – [15–35] years); Italic: *p*-Value; Italic bold: statistic significant *p*-Value

A higher prevalence of “no oral language” was found in the case group compared to the control group (*p* = 0.0001).

## Discussion

Since their first diagnostic application almost five decades ago, DBSs have been employed in many research areas and in clinical applications for several viruses, including Human immunodeficiency virus, Hepatitis C virus, Hepatitis B virus, Hepatitis A virus, Hepatitis E virus, Human cytomegalovirus, Epstein-Barr virus, Herpes simplex viruses, and measles-, dengue- and rubella-viruses. Although it is not expected that current wet sampling techniques will be replaced by the use of DBSs, this method allows sampling in individuals and settings with difficult access and/or a lack suitable storage facilities [13]. Indeed, the DBS method is a minimally invasive and more economic sampling method that is readily available and that facilitates sample collection and storage. It involves the collection of capillary blood from a fingerstick onto a protein-saver card, which is then air-dried and stored until processing. DBS samples can be stored and transported for testing at a later date, which may also provide enhanced surveillance in resource-limited settings [14].

In the current study, the accuracy of the IgG determination from DBS samples was assessed before the determination of rubella seroprevalence. The main limitations of this evaluation were the number of samples tested, which were lower than initially planned, and the fact that immunity by vaccination may not be similar to immunity by natural infection. In fact, children from São Tomé and Príncipe had higher average IgG results than Portuguese children (respectively 89.35 and 50.9; *p* = 0.034, data not presented). Despite these limitations, the results suggest that the determination of IgG for the rubella virus in DBS had a good correlation with the standard method. These results are in accordance with a previous publication that showed no significant differences in the antibody concentrations in paired serum-DBS samples, [15] although in our study, only the qualitative correlation is presented. Actually, quantitative DBS samples were also previously tested for the diagnosis of rubella during an outbreak, with an excellent correlation with the determination in serum, provided that the DBS indeterminate results were positive [16]. In addition, this methodology was used for the diagnosis of congenital rubella syndrome in another study, by detecting rubella IgM and IgG (the last in ≥ 6 months old infants) in DBS, although in this study, the comparison was not performed with the reference sample, serum, but with oral fluid samples [17]. Therefore, using DBS for serology determinations seems to be an appropriate and useful approach, particularly in countries where routine immunization is not performed, to estimate the rate of infection with the wild-type rubella virus.

The prevalence of IgG in the population from São Tomé and Príncipe of 303 individuals from 2 to 35 years of age was 66.7%, confirming that rubella infections are still very common in this country. In the group of children under 14 years of age, the prevalence of immunity to rubella was 50.3% (74/147 subjects), while in the age group between 15 and 35 years of age, the prevalence increased by up to 82.1% (128/156 subjects. This increase suggests that women of child-bearing age are still suffering from rubella primary infections, and 18% of these women are at risk for rubella infection during pregnancy and subsequent CRS/birth defects in their children.

Interestingly, an association between seroprevalence and SNHL was observed in the younger group (children <14 years, *p* = 0.025), but in the older group (>14 years, *p* = 0.528). While congenital rubella infection is a known cause of deafness, the relationship between postnatal acquired infection and hearing loss is not proven, although a few reports suggest that it may occasionally occur [18, 19]. In this study, it was not possible to conclude how many cases of HL are attributable to rubella infection because the time of infection cannot be determined. Therefore, the rate of positive IgG infection resulting from congenital infections is not known, although the probability of infection during the gestational period is higher in the youngest group. The detection of rubella IgM in DBS from newborns will help to clarify this key question. This will be part of a future project of our team.

The development of oral language is dependent of the hearing status. Therefore, we found an expected higher prevalence of “no oral language” in the case group than in the control group.

According to the CDC, the rubella vaccine may be administered in combination with the mumps vaccine or the measles and mumps vaccine [20]. These should be administered at 12 to 15 months of age, with a second dose given at 4 to 6 years of age. However, given the need to control the transmission of rubella during pregnancy, vaccination of female children between 10–12 years of age and women of childbearing age is also recommended. The rubella vaccine has been shown to be effective without significant side effects and should thus be quickly implemented in the population of São Tomé and Príncipe as well as other African countries in which the rubella vaccine is not currently implemented and where there is an increase of SNHL [4].

The combined vaccine with the measles and mumps vaccine would be advantageous because these two pathologies can also unleash SNHL in the course of the disease [20].

## Conclusion

Rubella is a preventable disease. Currently, most of the African countries do not use this vaccine.

Rubella infections are still very common in São Tomé and Príncipe, and women of child-bearing age are still at risk of rubella infection during pregnancy and subsequent CRS/birth defects of their children, justifying the urgency of vaccination against rubella.

According to the results obtained, a statistically significant association between the group of children under 14 years of age with SNHL and immunity for rubella was observed in this country. However, this study did not permit us to establish a cause-effect relationship between rubella infection and SNHL. Therefore, another study aiming to screen newborns for congenital rubella infection and to follow them for audiometric assessment is critical to determine the real impact of this infection in this African country.

## Abbreviations

CDC: Centre of Diseases Control
CI: Confidence interval
CRS: Congenital Rubella Syndrome
DBS: Dried blood spot
HL: Hearing loss
IMVF: Institute Marquês Valle Flor
OR: Odds ratio
SNHL: Sensorineural hearing loss
SPSS: Statistical package for the social sciences
WHO: World Health Organization
